# Impact on wine sales of removing the largest serving size by the glass: An A-B-A reversal trial in 21 pubs, bars, and restaurants in England

**DOI:** 10.1371/journal.pmed.1004313

**Published:** 2024-01-18

**Authors:** Eleni Mantzari, Minna Ventsel, Emily Pechey, Ilse Lee, Mark A. Pilling, Gareth J. Hollands, Theresa M. Marteau

**Affiliations:** 1 Behaviour and Health Research Unit, University of Cambridge, Cambridge, United Kingdom; 2 EPPI Centre, UCL Social Research Institute, University College London, London, United Kingdom; University of Toronto, CANADA

## Abstract

**Background:**

Interventions that alter aspects of the physical environments in which unhealthy behaviours occur have the potential to change behaviour at scale, i.e., across populations, and thereby decrease the risk of several diseases. One set of such interventions involves reducing serving sizes, which could reduce alcohol consumption. The effect of modifying the available range of serving sizes of wine in a real-world setting is unknown. We aimed to assess the impact on the volume of wine sold of removing the largest serving size by the glass from the options available in licensed premises.

**Methods and findings:**

The study was conducted between September 2021 and May 2022 in 21 licensed premises in England that sold wine by the glass in serving sizes greater than 125 ml (i.e., 175 ml or 250 ml) and used an electronic point of sale till system. It used an A-B-A reversal design, set over 3 four-weekly periods. “A” represented the nonintervention periods during which standard serving sizes were served and “B” the intervention period when the largest serving size for a glass of wine was removed from the existing range in each establishment: 250 ml (18 premises) or 175 ml (3 premises). The primary outcome was the daily volume of wine sold, extracted from sales data. Twenty-one premises completed the study, 20 of which did so per protocol and were included in the primary analysis. After adjusting for prespecified covariates, the intervention resulted in −420·8 millilitres (ml) (95% confidence intervals (CIs) −681·4 to −160·2 *p* = 0·002) or −7·6% (95% CI −12·3%, −2·9%) less wine being sold per day. There was no evidence that sales of beer and cider or total daily revenues changed but the study was not powered to detect differences in these outcomes. The main study limitation is that we were unable to assess the sales of other alcoholic drinks apart from wine, beer, and cider, estimated to comprise approximately 30% of alcoholic drinks sold in participating premises.

**Conclusions:**

Removing the largest serving size of wine by the glass from those available reduced the volume of wine sold. This promising intervention for decreasing alcohol consumption across populations merits consideration as part of alcohol licensing regulations.

**Trial registration:**

ISRCTN https://doi.org/10.1186/ISRCTN33169631; OSF https://osf.io/xkgdb.

## Author summary

**Why was this study done?:**

- Reducing the portion size of food reduces the amount of food people eat.
- Reducing the size of servings of alcoholic drinks sold by the glass in licensed premises could reduce alcohol consumption but there is no real-world evidence for this.

**What did the researchers do and find?:**

- We asked 21 licensed premises in England to remove the offer of their larger serving size of wine by the glass (usually 250 ml) from available options for 4 weeks. We compared the total daily volume of wine sold during the intervention period to that sold during nonintervention periods.
- Removing the largest serving size of wine by the glass (usually 250 ml) in licensed premises reduced the volume of wine sold by 7.6%. There was no evidence that it impacted sales of beer or cider, or total daily revenues.

**What do these findings mean?:**

- This intervention merits consideration for inclusion in alcohol licensing regulations.
- These findings are limited by our inability to assess the sales of cocktails and spirits and the potential for people to have compensated for reducing wine consumption by drinking more cocktails and spirits.

## Introduction

Alcohol consumption is the fifth largest contributor to premature death and disease globally [1]. In 2016, it was estimated to have caused approximately 3 million deaths worldwide and was responsible for 5.1% of the global burden of disease [2]. Reducing alcohol consumption across populations is a global public health priority [3]. This is reflected in the World Health Organization’s (WHO) SAFER initiative (https://www.who.int/initiatives/SAFER), launched in 2018 with the aim of helping governments reduce harmful alcohol consumption and its related consequences [4]. This global priority is also reflected in WHO Europe’s recent decision to commit all member states to a comprehensive plan for accelerating action on reducing alcohol consumption across the continent [5].

Many cues in physical and economic environments influence alcohol consumption across populations. These include advertising, marketing [6–9], product labelling [10–12], the availability of alcohol [13–17], and price [18,19]. Interventions, therefore, that target cues in physical and economic environments have significant potential, based on indirect evidence, to exert effects scalable to populations [20,21]. Most of the focus to date has been on interventions that increase the price of alcoholic drinks, and control their marketing and licensing [22,23]. Although these interventions are effective at reducing consumption across populations, more interventions are needed to reduce consumption further. One set of promising interventions involves reducing the portion or serving sizes of products that harm health. When presented with smaller portions, packages, or related tableware, such as plates or glasses, people consume less [24]. While a small minority of people use smaller serving sizes to regulate their alcohol consumption [25], this well-documented “portion size effect” for food has—until recently—been neglected as a focus of study in relation to alcoholic drinks and its potential as an alcohol control policy.

Reducing the size of containers, including the glasses and bottles in which alcohol is packaged and served has the potential to reduce alcohol consumption. Larger 370 ml wine glasses increased the volume of wine sold, and therefore consumed, compared to 300 ml glasses in restaurants by approximately 7% [26], while in homes, smaller 290 ml wine glasses reduced the amount of wine drunk by around 6·5% compared with 350 ml glasses [27]. Bottle size may also influence alcohol consumption. Drinking wine at home from 50 cl bottles, compared with 75 cl bottles, reduced the amount consumed by 4·5% [28]. In a subsequent study assessing drinking at home from 37·5 cl compared with 75 cl bottles, a smaller and less certain reduction of 3·6% was observed [27].

Reducing the serving sizes of alcoholic drinks available in licensed premises could also reduce consumption. Two studies, 1 conducted in a laboratory and 1 in a semi-naturalistic context of a pub in which drinks were served in sizes predetermined by the researchers, found a reduction in alcohol consumed on a single occasion when serving sizes were smaller [29]. In the first of these, participants were randomised to 1 of 2 groups and served either larger serving sizes of cider (460 ml), lager (460 ml) or wine (165 ml), or smaller serving sizes (cider/lager: 345 ml; wine: 125 ml). Alcohol consumption was 20% lower in the group served small sizes. In the second study, participants were recruited to the study to attend 1 of 4 quiz nights during which they were randomised either to the offer of pints (568 ml) of beer/cider and 175 ml glasses of wine, or to the offer of serving sizes reduced by 33%, i.e., 2/3 pints (379 ml) for beer/cider and 125 ml for wine. Sales were reduced by 28% when serving sizes were smaller. However, the impact of offering reduced serving sizes has yet to be evaluated in real-world settings.

The current study is the first to our knowledge to be conducted in a real-world field setting of licensed premises operating commercially. The study targeted wine consumption, given wine is the most commonly drunk alcoholic beverage in Europe, including the United Kingdom, where over a third of the population prefers wine over other alcoholic beverages, such as beer [30]. It was designed to estimate the impact on the volume of wine sold of removing the largest serving size by the glass from the options available. We hypothesised that removing the largest available serving of wine by the glass would reduce the volume of wine sold.

The secondary aims of the study were to assess the impact of the intervention on: (i) the volume of wine sold in different serving sizes, in order to explore the serving sizes of choice in the absence of the largest serving of wine by the glass; (ii) the volume of beer and cider sold, in order to assess whether the absence of the largest serving of wine led to a shift in beer and cider consumption; and (iii) total revenue, to explore whether removal of the largest serving of wine had an impact on earnings.

## Methods

### Ethics statement

The study was approved by the University of Cambridge Psychology Research Ethics Committee (reference no: PRE.2019.035). The study protocol (S1 Study protocol) was preregistered (ISRCTN: ISRCTN33169631 https://doi.org/10.1186/ISRCTN33169631; Open Science Framework: registration https://osf.io/xkgdb/; protocol: https://osf.io/sxe9t; statistical analysis plan (S1 Statistical Analysis Plan): https://osf.io/6n9xh).

### Study design

The study used an A-B-A treatment reversal design comprising 3 consecutive four-week periods in which “A” represented the nonintervention periods during which standard serving sizes were available, and “B” represented the intervention period during which the largest serving size of wine by the glass available in that establishment (either 250 ml or 175 ml) was removed from sale.

### Setting

The study was conducted in pubs, bars, and restaurants.

### Participants

Participants were 21 licensed premises in England. Their location and other characteristics are shown in Table 1. The majority of these (86%) were pubs, located in London (62%), in more deprived areas, as determined by their IMD (index of multiple deprivation) scores, with 76% of premises located in the first and second most deprived quintiles of the city.

To be eligible to take part in the study, licensed premises had to meet the following criteria:

i. sell wine by the glass in serving sizes greater than 125 ml (i.e., 175 ml or 250 ml)
ii. sell a minimum of 100 glasses of wine on average per week
iii. be willing to remove the largest serving size for a glass of wine
iv. have an electronic point of sale (EPOS) till system to record daily sales of all drinks and their served sizes
v. be primarily indoor, permanent establishments in a fixed location; i.e., not purposefully temporary or time-limited (e.g., pop-up), or mobile venues (e.g., vans).

**Table 1:** Characteristics of recruited licensed premises.

Premises number	Location	Index of multiple deprivation quintile*	Premises type**	Baseline daily revenue (£) (mean (sd))	Largest serving of wine by glass***
1	Hackney, London	2	Pub	1,327·8 (1,245·9)	250 ml
2	South Cambridgeshire	5	Pub	898·6 (372·4)	250 ml
3	Stroud, Gloucester	5	Pub	1,471·9 (555·1)	250 ml
4	Stroud, Gloucester	3	Pub	846·0 (550·8)	250 ml
5	Lewisham, London	2	Pub	2,599·6 (1,288·6)	250 ml
6	Islington, London	4	Pub	2,795·0 (1,690·6)	175 ml
7	Lewisham, London	2	Pub	2,724·1 (1,231·2)	250 ml
8	Hackney, London	2	Pub	2,672·1 (2,064·6)	250 ml
9	Islington, London	2	Pub	1,776·6 (1,152·6)	250 ml
10	Eastleigh, Southampton	5	Pub	1,422·6 (1,318·6)	250 ml
11	Hackney, London	2	Pub	941·1 (765·9)	250 ml
12	Brighton and Hove	3	Pub	1,181·0 (642·4)	250 ml
13	Lambeth, London	2	Pub	4,504·9 (1,693·2)	250 ml
14	Southwark, London	3	Pub	4,840·2 (2,795·0)	250 ml
15	Southwark, London	2	Pub	3,841·1 (1,278·8)	250 ml
16	Southwark, London	2	Bar	3,553·0 (2,993·6)	250 ml
17	Brighton and Hove	1	Pub	1,789·5 (1,106·4)	175 ml
18	Brighton and Hove	1	Restaurant and Bar	981·9 (10,061·0)	250 ml
19	Brighton and Hove	2	Pub	520·9 (484·1)	250 ml
20	Greenwich, London	2	Pub	1,015·0 (526·6)	250 ml
21	Hammersmith and Fulham, London	4	Champagne and Cocktail Bar	1,671·1 (2,014·9)	175 ml

*1 = most deprived; 5 = least deprived.

** Description of premises type taken from each premises’ website.

*** A 250 ml glass of wine contains on average 2.5 standard drinks or 3 units and a 175 ml glass of wine contains on average 1.7 standard drinks or 2.3 units.

**Fig 1:**
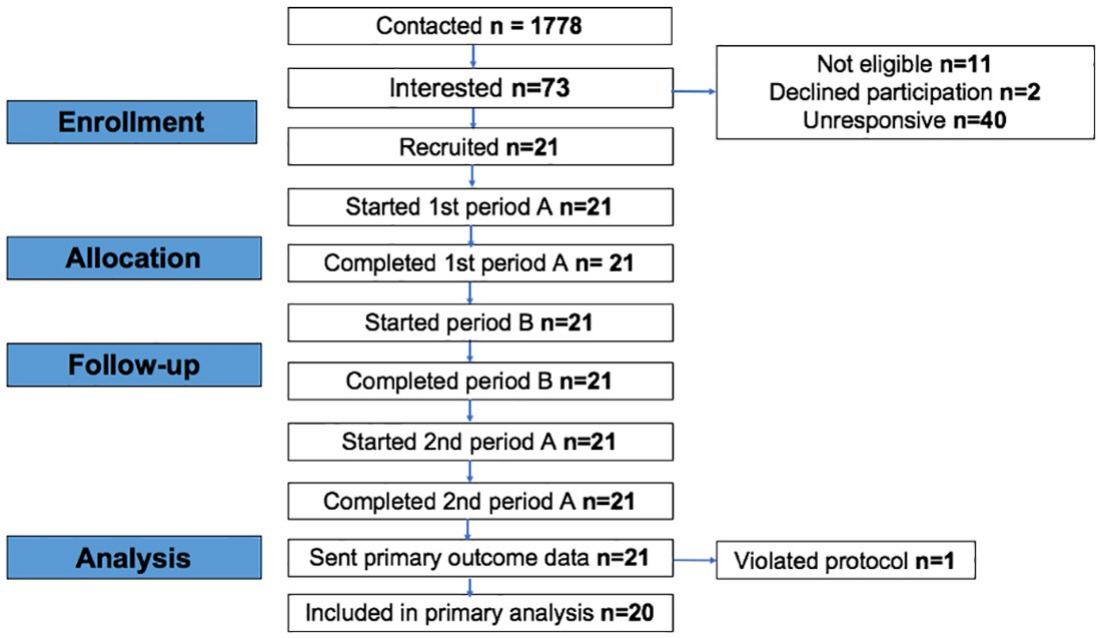
CONSORT diagram of flow of premises through study.

The flow of premises through the study is shown in Fig 1. Twenty-one licensed premises were recruited from 1,778 that were contacted in targeted geographical areas, a recruitment rate of just over 1%.

#### Sample size calculation

The current study was initially planned with a minimum of 5 licensed premises. Based on available resources, recruitment was increased to 21 licensed premises. There was prior data from a pilot study in 2 licensed premises—both student bars—using an A-B-A design with each period lasting 4 weeks. Based on a linear mixed effects model of the daily data for the 2 premises, power simulations [31] suggested that 85 premises were need to be recruited to provide at least 80% power to detect a predicted effect of −226·8 ml wine sold during the intervention. It was not feasible to recruit this number of premises. Given these simulations, power to detect possible effects with 21 establishments was expected to be low. The study was therefore considered opportunistic, providing preliminary evidence to inform future research.

### Intervention

Licensed premises reduced their range of serving sizes for glasses of wine by removing the largest serving from their available options. This was either 250 ml or 175 ml, with 125 ml sizes always available in keeping with current regulations for selling alcohol in licensed premises. Eighteen premises offered 250 ml, 175 ml, and 125 ml servings during their nonintervention periods. During the intervention, these premises offered only 175 ml and 125 ml servings. Three premises offered 175 ml and 125 ml servings during their nonintervention periods and only 125 ml servings during the intervention. Menus and signage were updated to reflect changes. Sizes of bottles and carafes remained unchanged.

Within the TIPPME intervention typology for changing environments to change behaviour [21], the type of intervention used in the current study was “Size,” and focused on the “Product” itself, i.e., the alcoholic drink (as opposed, for example, to aspects of the wider environment).

### Measures

#### Primary outcome

Daily volume (in ml) of all wine sold (by the glass, as well as sales by the bottle and carafe, if offered), extracted from electronic records of sales.

#### Secondary outcomes

The following outcomes were extracted from electronic records of sales:

i. Daily volume (in ml) of wine sold by each serving size:
- 125 ml
- 175 ml
- 250 ml
- 500 ml carafe
- 750 ml bottle
- 1,000 ml carafe
ii. Daily volume (in ml) of beer and cider sold
iii. Daily revenue (in £) from food, alcoholic, and nonalcoholic drinks.

Note that information on spirits and other alcoholic drinks could not be extracted from sales reports. Cocktails and spirits are estimated to have comprised approximately 30% of alcoholic drinks sold in participating premises [32].

#### Covariates

Given that daily temperature, day of the week, season, and holidays can influence alcohol sales [33,34], the following covariates were considered:

i. Maximum daily local temperature
ii. Special events (e.g., Bank Holidays, other holidays, major sporting events)
iii. Total revenue
iv. Day of the week
v. Study day from start of a period
vi. Season at start of study: autumn or winter.

### Procedure

Potentially eligible pubs, bars, and restaurants were identified through a publicly available database (www.whatpub.com). Those based in 1 of 8 geographical areas were invited via email to participate in the study. These areas were selected so that either the research team or collaborators could readily travel there to conducted fidelity checks. Those interested in taking part were sent more information about the study and were assessed for eligibility over the telephone. Eligible premises wishing to participate provided written informed consent for taking part. Consent from individual consumers was not deemed necessary as individual-level data were not collected.

Recruited premises changed their available serving sizes for wine on 2 occasions over a period of 12 weeks, first to remove their largest serving size by the glass during the intervention period (B), and second to return it during the second nonintervention period (A). Till systems, menus, and signs were updated as appropriate to reflect the available serving sizes. Premises leads were contacted 1 day before each change to remind them of the required change. Premises leads and staff were asked not to mention the study to any patrons who asked about the serving size changes, being given a simple explanation in the event of such an inquiry: “We have been receiving requests for differently sized drinks, so we are trying out some changes for a few weeks.”

Fidelity to the protocol was checked twice to establish whether the correct serving sizes were on offer during the intervention period (B) and subsequent return to nonintervention period (A). Checks were conducted during the first week of each study period. No premises failed any fidelity checks.

Data were collected between September 2021 and May 2022. Premises were paid £1,000 (plus 20% value added tax (VAT)), later increased to £1,500 (plus 20% VAT) to help increase the recruitment rate, for taking part in the study and providing all requested data. Premises were also reimbursed for the costs of any necessary changes to menus and signs.

### Data analysis

Premises provided daily reports that listed sales of each individual product. The format of the reports differed between premises. The data cleaning process involved aggregating sales according to product type (i.e., beer and wine) and serving size. Analyses were performed on R (4.0.3). Unadjusted summaries of the volume of wine sold during the nonintervention and intervention periods were calculated both overall and for each specific serving size. Outliers in the daily data were identified using range checks, scatter plots, median absolute deviation values, and histograms on daily data, for further checking that these were true values. The potential outliers identified were all deemed genuine values and it was assumed that the model covariates (total revenue—proxy for site busyness—and specials events) could handle these to ensure no outliers in the model residual diagnostics.

#### Primary analysis

A generalised linear mixed model (generalised additive models that can accommodate heterogeneity) was used to estimate daily volume of wine sales, the primary outcome, according to study period (A versus B). Premises were treated as a random factor and heterogeneity (in terms of size and other characteristics which result in different amounts of variability) between premises was modelled. The analysis included prespecified covariates for day of the week, study day (number ranging from 1 to 84, to allow for potential linear trends over time), and total revenue from all food and drink (as a proxy for premises busyness). An overall effect was estimated from this model. The mean difference and associated 95% confidence intervals (CIs) and *p*-value, as well as a Cohen’s d effect size and its 95% CI were calculated. All regression model diagnostics (residual plots, worm plots) were checked and were satisfactory.

Only premises that met the following 3 conditions were included in the primary analysis:

i) completed the study in full, i.e., all 12 weeks
ii) provided primary outcome data for the 12 weeks of the study
iii) adhered to the protocol for intervention implementation, i.e., they passed the fidelity checks and their data did not suggest that the largest serving size of wine by the glass was sold during Period B.

#### Sensitivity analyses

Four sets of sensitivity analyses were conducted to check the robustness of the primary analysis. For each, an overall effect was estimated, as well as mean differences and associated 95% CIs and *p*-values:

1. Regression analysis, repeating the primary analysis but taking into account 3 additional covariates: (i) the total number of special events in each period; (ii) season at the start of the study (autumn or winter); (iii) maximum daily local temperature.
2. Regression analysis, repeating the primary analysis but adding daily-level data from all premises, including those that violated the protocol for intervention implementation.
3. Regression analysis, repeating the primary analysis but including the 2 nonintervention periods as separate factor levels (i.e., using A1, B, and A2 levels for the periods).
4. As data might be less variable when aggregated at the period level, a regression analysis was conducted using period-level data to compare mean daily sales during period A (aggregate value for 2 four-week A period) and mean daily sales during period B (aggregate value for 1 four-week B period). Mean daily sales for each period were calculated by adding the total volume of wine sold and dividing by the number of days the premises were open during each “A” and “B” period. No covariates were included in this analysis due to the inclusions of only 2 data points per site (i.e., aggregate of A periods and aggregate of B).

#### Secondary analyses

For the secondary outcomes generalised linear mixed models were used, with the distribution of the data assessed by model diagnostics dictating which model was most appropriate (e.g., Poisson regression). For each, an overall effect was estimated, as well as mean differences and associated 95% CIs and *p*-values:

The following secondary analyses were conducted:

1. Regression analyses to estimate the number of wine drinks sold in each serving size (125 ml, 175 ml, 250 ml, 500 ml carafes, 750 ml bottle, 1000 ml carafes) according to the study Period (A versus B).
2. A regression analysis to estimate the daily volume of beer and cider sold according to the study Period (A versus B). The analysis included covariates for day of the week, study day (number ranging from 1 to 84), and total revenue from all food and drink.
3. A regression analysis to estimate total revenue from all food and drink according to the study Period (A versus B). The analysis included covariates for day of the week and study day (number ranging from 1 to 84).

## Results

One premises was excluded from the primary analysis for violating the protocol and selling the largest serving of wine by the glass during the intervention period, as identified by inspection of the data.

### Primary outcome: Volume of wine sales

**Fig 2:**
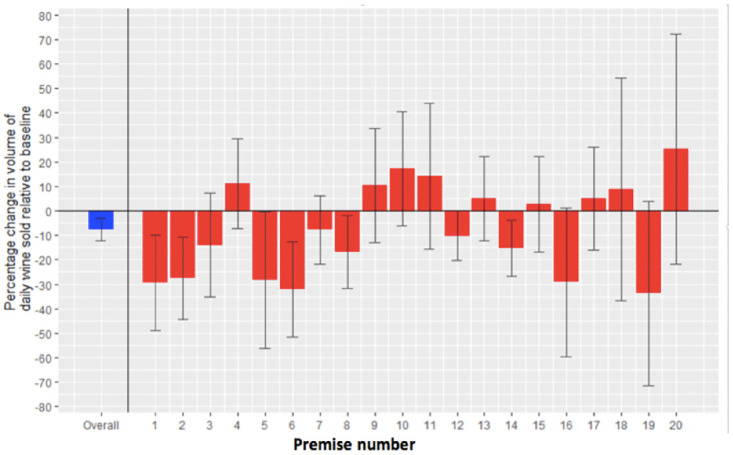
Predicted change in wine sold (% (95% CI)) after removing the largest serving sizes by the glass, derived from the generalised additive linear mixed model.

The unadjusted mean daily volume of wine sold per premises during the nonintervention periods (A) was 5,198·7 ml (sd = 5,021·9) and 4,814·1 ml (sd = 5,188·4) during the intervention period (B). After accounting for prespecified covariates (day of the week; study day; total revenue), there was a significant effect of study period, with −420·8 ml (95% CI −681·4 to −160·2) or −7·6% (95% CI −12·3%, −2·9%) less wine sold per day during the intervention period (B) compared to the 2 nonintervention periods (A) (Table 2). There was heterogeneity between premises (sigma coefficients were statistically significant at *p* < 0·001 (Table 3)). Fig 2 shows the effect of the intervention on wine sales overall and for each individual premises.

**Table 2:** Mixed effects generalised additive linear mixed model (GAM) main results (95% CI) estimating the volume (ml) of wine sold per day (*n* = 20).

				95% CI for estimate	
	Estimate (SE)	t-value	*P*-value	Lower	Upper
Intercept	856·06 (208·12)	4·11	<0·001	448·1	1,263·9
Study period (ref: nonintervention)	−420 79 (132 96)	−3·16	0·002**	−681·4	−160.2
Day of the week_Tuesday (ref: Monday)	540·92 (238·34)	2·27	0·024*	73·4	1,008·1
Day of the week_Wednesday (ref: Monday)	833.23 (235·23)	3·54	<0·001**	372·2	1,294·3
Day of the week_Thursday (ref: Monday)	928·55 (238·16)	3·89	<0·001**	461·8	1,395·3
Day of the week_Friday (ref: Monday)	1,160·84 (249·29)	4·66	<0·001**	672·2	1,649·5
Day of the week_Saturday (ref: Monday)	364·94 (243·92)	1·49	0·135	−113·1	843·0
Day of the week_Sunday (ref: Monday)	900·88 (239·29)	3·76	<0·001**	431·9	1,369·9
Study day	−1·70 (2·62)	−0·65	0·517	−0·68	3·44
Total revenue	1·84 (0·047)	38·79	<0·001	1·75	1·94

*Significant at the *p* < 0·05 level.

**Significant at the *p* < 0·01 level.

CI, confidence interval; SE, standard error.

**Table 3:** Mixed effects generalised additive linear mixed model (GAM) variance estimates from estimating the volume (ml) of wine sold per day for each individual licensed premises (reference: Premises 1).

Modelling of the variance (log link):	Estimate (SE)	t-value	*P*-value
Intercept	0.474 (0.079)	5.96	<0.001
Premises 2	0.059 (0.113)	0.52	0.600
Premises 3	0.384 (0.115)	3.32	0.001**
Premises 4	−0.006 (0.117)	−0.05	0.953
Premises 5	1.014 (0.111)	9.12	<0.001**
Premises 6	0.457 (0.110)	4.14	<0.001**
Premises 7	0.372 (0.113)	3.29	0.001**
Premises 8	0.262 (0.113)	2.31	0.020
Premises 9	0.404 (0.110)	3.67	<0.001**
Premises 10	0.081 (0.113)	0.71	0.474
Premises 11	0.542 (0.112)	4.83	<0.001**
Premises 12	0.938 (0.111)	8.41	<0.001**
Premises 13	1.05 (0.115)	9.15	<0.001**
Premises 14	0.562 (0.110)	5.10	<0.001**
Premises 15	0.893 (0.121)	7.34	<0.001**
Premises 16	0.508 (0.112)	4.53	<0.001**
Premises 17	0.702 (0.131)	5.37	<0.001**
Premises 18	0.404 (0.118)	3.41	<0.001**
Premises 19	0.939 (0.110)	8.50	<0.001**
Premises 20	0.953 (0.118)	8.06	<0.001**

*Significant at the *p* < 0.05 level.

**Significant at the *p* < 0.01 level.

CI, confidence interval; SE, standard error.

### Sensitivity analyses

Results and conclusions were unchanged when performing an intention-to-treat analysis (*n* = 21) that included the one premises that had violated the protocol, with −424·8 ml (95% CI −679·9 to −169·8, *p* = 0·001) or −7·6% (95% CI −12·2% to −3·1%) less wine being sold per day during the intervention period (B) compared to the nonintervention periods (A) (S1 Table).

Including 3 additional covariates in the model (total number of special events such as Bank Holidays in each period; season at the start of the study; maximum daily local temperature) also had no effect on the main result. The model showed that −418·6 ml (95% CI −675.7 to −161·6, *p* = 0·001) or −7·5% (95% CI −12·2% to −2·9%) less wine was sold per day during the intervention period (B) compared to the nonintervention periods (A) (S2 Table).

Using period-level data (i.e., an aggregate value for sales during the nonintervention periods and an aggregate value for the intervention period) rather than daily-level data, generated results that were consistent with the conclusion that wine sales were lower during the intervention period (B) compared to the nonintervention period (A) (−306·3 ml; 95% CI −804·9 to 192·3) but the difference was not statistically significant (*p* = 0·233).

In order to assess whether the 2 nonintervention periods were comparable, an additional analysis was conducted in which the 2 nonintervention periods were added to the model separately. The results showed that sales of wine did not significantly differ during the 2 nonintervention periods (A) (−85 ml less wine per day was sold during the second nonintervention period compared to the first; 95% CI −99·6 to 82·5; *p* = 0·854) (S3 Table), justifying the modelling choice of combining data from both nonintervention periods for the primary analysis.

### Secondary outcomes

#### Wine sales by serving size

The unadjusted mean daily volume of wine sold in 125 ml serving sizes during the nonintervention periods (A) was 351·1 ml (sd = 450·7) and 583·5 ml (sd = 926·7) during the intervention period (B). A Poisson regression showed that the number of glasses of wine sold in serving sizes of 125 ml increased during the intervention compared to the nonintervention periods (incident rate ratio (IRR) = 1·64; 0·49 95% CI 0·44 to 0·55; *p* < 0.001).

The unadjusted mean daily volume of wine sold in 175 ml serving sizes during the nonintervention periods (A) was 1,487·9 ml (sd = 1,418·2) and 2,372·6 ml (sd = 2,455·5) during the intervention period (B). A Poisson regression showed that the number of glasses of wine sold in serving sizes of 175 ml increased during the intervention compared to the nonintervention periods (IRR = 1·54; 0·43 95% CI 0·40 to 0·46; *p* < 0·001).

The unadjusted mean daily volume of wine sold in 500 ml carafes during the nonintervention periods (A) was 116·9 ml (sd = 523·6) and 160·7 ml (sd = 1,128·9) during the intervention period (B). There was no evidence of a difference in the number of 500 ml carafes of wine sold during the intervention compared to the nonintervention periods (0·07 95% CI −0·13 to 0·27; *p* = 0·49).

The unadjusted mean daily volume of wine sold in 750 ml bottles during the nonintervention periods (A) was 1,623·7 ml (sd = 2,829·8) and 1,687·5 ml (sd = 2,930·9) during the intervention period (B). There was no evidence of a difference in the number of 750 ml bottles of wine sold during the intervention compared to the nonintervention periods (−0·01 95% CI −0·08 to 0·07; *p* = 0·73).

The unadjusted mean daily volume of wine sold in 1,000 ml carafes during the nonintervention periods (A) was 9·35 ml (sd = 133·3) and 6·80 ml (sd = 100·9) during the intervention period (B). There was no evidence of a difference in the number 1,000 ml carafes of wine sold during the intervention compared to the nonintervention periods (0·17 95% CI −1·08 to 1·43; *p* = 0·79).

#### Volume of beer and cider sales

The unadjusted mean daily volume of beer and cider sold during the nonintervention periods (A) was 1,002,500 ml (sd = 1,008,900) and 1,005,900 ml (sd = 1,219,700) during the intervention period (B). There was no evidence of a difference in the volume (ml) of beer and cider sold per day between the intervention and nonintervention periods (306·74 ml 95% CI −1,355·2 to 1,968·7; *p* = 0·72).

#### Revenue

The unadjusted mean daily revenue during the nonintervention periods (A) was £2,051·6 (sd = 1,824·1) and £2,089·1 (sd = 2,058·9) during the intervention period (B). There was no evidence of a difference in total daily revenue (£) between the intervention and nonintervention periods (−1·90, 95% CI −63·37 to 59·56; *p* = 0·95).

## Discussion

Removing the largest serving size of wine by the glass from the range of options available in licensed premises, after controlling for other factors, reduced the volume of wine sold by 7·6%. Sales of the smaller serving sizes of wine by the glass—125 ml and 175 ml—were increased. There was no evidence of a change in sales of wine by the bottle and sales of beer and cider or a change in daily revenues but the study was not powered to detect differences in these outcomes.

### Findings in context

Removing the largest serving size of wine by the glass in licensed premises had the hypothesised effect of reducing the volume of wine sold. This is in keeping with the wealth of literature assessing the impact of smaller serving sizes on food consumption [24] and the very limited literature on alcohol consumption, comprising 2 studies conducted in semi-naturalistic contexts [29].

The results of the current study suggest that when the largest serving size of wine by the glass (typically 250 ml) was not available, people shifted towards the smaller options (125 ml and 175 ml) and neither drank the equivalent amount of wine nor more, for example, by opting to buy wine by the carafe or bottle. The increase in sales was slightly larger for 125 ml servings compared to 175 ml serving. Given that in most premises the largest serving was 250 ml, this implies that people did not automatically choose the next available size (175 ml). It is not clear why this was the case. One possibility is that when customers who had planned to drink a large glass of wine (250 ml) were told it was not available, they planned to drink two 125 ml services but stopped after one. People have the tendency to consume a specific number of “units” (e.g., number of glasses or bottles, number of cookies or slices of cake), regardless of portion or package size [35]. This helps explain why smaller serving sizes reduce alcohol consumption: people tend to order a pre-set number of glasses, and with less alcohol in each glass they drink less overall.

The current study found no evidence that the intervention affected beer and cider sales, suggesting people did not compensate for their reduced wine consumption by drinking more of these alcoholic drinks. Importantly, there was also no evidence that the intervention affected total daily revenues, implying that participating licensed premises did not lose money as a result of removing the largest serving size for glasses of wine. This might reflect the pricing of glasses of wine, with 125 ml servings usually having a higher profit margin than 250 ml glasses [36]. Important to note is that the study was not powered to provide statistically meaningful data on secondary outcomes.

### Strengths and limitations

To our knowledge, this is the first study to estimate the impact on sales—a proxy for consumption—of removing the largest serving of sizes of alcoholic drinks in pubs, bars, and restaurants. Further strengths include features of the design that reduce the risk of bias, including the use of objective measures to assess the primary and secondary outcomes, i.e., electronic records of sales, as well as the large number of participating premises and the high retention rate.

The study has several limitations. First, due to the complexity of the sales reports provided by participating premises, it was not possible to assess the sales of all alcoholic drinks. While we could reliably assess sales of wines, beers, and ciders—estimated to make up more than 70% of alcoholic drinks in licensed premises in the UK [32]—we were unable to assess sales of spirits or cocktails. It is not known, therefore, whether people compensated for reduced wine consumption by drinking more of these other alcoholic drinks. It is also not known whether customers compensated for the reduced serving sizes by drinking wines higher in % alcohol by volume (ABV). This possibility, however, is minimal given that there were very small variations in the % ABV of wines sold by premises, ranging from 12% to 14%, information very rarely mentioned in wine lists anyway. Also, wine lists did not change during the study and none of the premise managers interviewed at the end of the study mentioned any changes in the drinking patterns of their customers during the intervention period relating to wine strength. Second, we were also unable to control for the number of patrons visiting the premises during each study period, to assess whether differences in sales could be attributed to differences in how busy premises were. We attempted to take this into consideration by using total revenue as a proxy measure of busyness. Third, the majority of premises were in London and constituted a small proportion of those approached, which potentially restricts the generalisability of the findings. Fourth, the study used an A-B-A reversal design, which has a higher risk of bias than an experimental design, although the analyses accounted for notable events including public holidays and temperature fluctuations, which may have confounded the effects. Fifth, although outcomes were assessed using objective measures, they concerned sales rather than actual consumption. Measuring consumption directly at scale in these kinds of real-world settings is not feasible. Sales are, however, a valid—as well as practicable—proxy for consumption [37] and are commonly used in behavioural research [38–40]. Finally, the impact of removing the largest serving size of wine was assessed only during a four-week period. Whether observed effects are sustained over time remains to be assessed in future research.

### Implications for research and policy

Although the intervention resulted in a relatively small reduction in the volume of wine sold by each premises (7.6% or 421 ml per day, equivalent to approximately 4 standard drinks or 5 units per premises), given that no level of alcohol consumption is currently considered safe for health with even light and moderate consumption contributing to the development of many cancers [41], such a reduction could meaningfully contribute to population health. In England, legal serving sizes of wine by the glass are 125 ml, 175 ml, and multiple of these sizes [42]. It is a requirement that the smallest size—125 ml—is offered to customers but there is no restriction on the largest serving size that can be offered. Restricting the sale of the largest serving of wine by the glass (250 ml) in licensed premises—similarly to the ban proposed by the mayor of New York City in 2012 on serving sizes of sugary drinks larger than 16 ounces [43]—could contribute to policies for reducing alcohol consumption at the population level and merits consideration as part of alcohol licensing regulations.

This is the first study to assess the impact of this intervention. The findings, therefore, require replication in future studies. If effects are replicated, it would strengthen the case for a such consideration.

It is unknown whether similar effects would result from removing the largest serving size for other types of alcoholic drinks including beer, as no real-world studies assessing this exist. We attempted to conduct such a study to estimate the impact of removing the largest serving size for beer—usually a pint (568 ml)—and replacing it with a two-thirds measure, but were unable to find any pubs, bars, or restaurants willing to do this from almost 2,000 contacted. This likely reflects that the pint has been the customary serving size for beer in the UK for centuries [44]. In contrast, it was not until relatively recently that licensed premises started serving 250 ml glasses of wine. Smaller glasses containing 125 ml wine were once considered the standard size for serving wine by the glass [45]. In the UK, this default has now been replaced with the 175 ml measure. The size of wine glasses has also increased in recent years, almost doubling in the last thirty years [46], and likely contributing to an increase in wine consumption [26].

Regulating serving sizes in licensed premises could help shift social norms for what constitutes an appropriate serving size [47], both for consumption out of the home, such as in pubs and bars, and for consumption at homes where most drinking occurs [48]. This possible indirect effect of the intervention awaits study. Whether regulating serving sizes interacts with existing alcohol controls concerning the pricing, marketing, and licensing regulations [22,23]—also awaits study.

Interventions that reduce serving or package sizes are generally less supported by the public than information-based interventions, such as health warning labels [49–51]. In the current study, managers reported receiving few complaints from customers when the largest serving size was removed (4/21 premises reported receiving some complaints). Although the intervention would potentially be acceptable to premises’ managers given there is no evidence that it can result in a loss in sales, were it to be implemented as part of alcohol control policies it would likely lead to opposition from the alcohol industry, given its potential to reduce sales of targeted drinks [52]. The impact of such opposition on policy-makers will in part be modified by the level of public support for the intervention [53,54]. Public support for this and indeed a range of policies in health and other domains is sensitive to evidence of the policy’s effectiveness [55,56]. Communicating the effectiveness of a policy to achieve a valued outcome increases its public support. While we are unaware of any studies examining this in the context of alcohol serving sizes, a policy that increased the price of alcohol by introducing a minimum unit price of £1 was supported by 63% of a representative sample of the English population when informed of its effectiveness at reducing crime and hospital admissions, compared with 43% when not given this information [57]. Research is needed to explore the acceptability of sizing interventions for reducing alcohol consumption, as well as methods for increasing low levels of public support.

### Conclusion

Removing the largest serving of wine by the glass from the range of options available in licensed premises reduced the volume of wine sold. This suggests this is a promising intervention for decreasing alcohol consumption across populations, which merits consideration as part of alcohol licensing regulations.

## Supporting information

S1 CONSORT Checklist CONSORT checklist of information relating to study. (DOC)

S1 Study protocol The impact of altering serving sizes of beer and wine on alcohol consumption: A field study. (DOCX)

S1 Statistical analysis plan The impact of altering serving sizes of wine on alcohol consumption: ANALYSIS PLAN for Intervention 2 (wine study). (DOCX)

S1 Table Mixed effects GAM regression estimates (95% CI) for volume (ml) of wine sold per day (*n* = 21)—intention to treat analysis. (DOCX)

S2 Table Mixed effects GAM regression estimates (95% CI) for volume (ml) of wine sold per day (*n* = 20)—additional covariates. (DOCX)

S3 Table Mixed effects GAM regression estimates (95% CI) for volume (ml) of wine sold per day (*n* = 20)—separating nonintervention periods. (DOCX)

## Abbreviations

ABV: alcohol by volume
CI: confidence interval
CL: centilitre
EPOS: electronic point of sale
IMD: index of multiple deprivation
IRR: incident rate ratio
ML: millilitre
VAT: value added tax
WHO: World Health Organization
