# Supplementary material for: Impact on wine sales of removing the largest serving size by the glass: An A-B-A reversal trial in 21 pubs, bars, and restaurants in England
Source: PLoS Med. 2024 Jan 18;21(1):e1004313. doi: 10.1371/journal.pmed.1004313 (PMC10796003; doi:10.1371/journal.pmed.1004313)
Supplement: S1 Study protocol — (DOCX) [file pmed.1004313.s002.docx]

STUDY PROTOCOL

The impact of altering serving sizes of beer and wine on alcohol consumption: a field study

Eleni Mantzari, Emily Pechey, Ilse Lee, Katie De-loyde, Mark Pilling*,* Gareth J. Hollands, Theresa M. Marteau*

# Abstract

## Background

Few studies have assessed the impact of modifying the range of serving sizes of beer or wine on alcohol sales in licensed premises. This study assesses the impact of two distinct interventions on alcohol sales: 1. Adding a two-third pint option for beer and cider sold on tap; 2. Removing the largest serving size for a glass of wine.

## Methods

A minimum of fourteen sites will be recruited to add the two-third pint option - Intervention 1 - and a minimum of five sites will be recruited to remove their largest wine serving size for a glass of wine - Intervention 2. Sites will be recruited to implement either one or both of the interventions. If the latter, these will be implemented sequentially.

For Intervention 1, sites will introduce an additional two-third pint option for all beer and cider available on tap, priced proportionately.

For Intervention 2, sites will remove the largest serving size for a glass of wine from the existing range (250ml or 175ml).

A reversal design will be used for each intervention with serving sizes changed twice over three four-week periods where A is the control period and B is the intervention period (i.e. ABA).

The primary outcome is the daily volume of beer and cider or wine sold.

## Discussion

The results of this study will provide evidence for the potential of reducing the volume of alcohol consumed through modifying the range of serving sizes of beer and wine in licenced premises.

*Author for correspondence: Theresa Marteau, [tm388@medschl.cam.ac.uk](mailto:tm388@medschl.cam.ac.uk)

# Background

Alcohol is the fifth largest contributor to premature death in high-income countries worldwide (Gakidou et al., 2017), causing an estimated loss of 167,000 working years in England in 2015 (Burton et al., 2017).

There is growing recognition of the potential for interventions that change physical micro-environments to reduce harmful health-related behaviours at the population level (e.g. Hollands et al., 2013). Reducing the size of portions, packaging and tableware decreases consumption of food and non-alcoholic beverages (Hollands et al., 2015). There is also evidence that altering the size of containers in which alcohol is packaged, sold and served can reduce consumption. Specifically, smaller wine bottles can reduce the amount consumed in homes (Codling, Mantzari et al. 2020), and larger wine glasses can increase the volume of wine sold, and therefore consumed, in restaurants (Pilling, Clarke et al. 2020). However, there is a lack of evidence for the effect of modifying the range of available serving sizes of alcoholic beverages on alcohol consumption, either by removing the largest serving size available, or introducing a smaller serving size to an existing range. Kersbergen and colleagues (2018) found that reducing the serving sizes of beer and wine decreased alcohol consumption in both a laboratory and field setting. A limitation of the field study is that it examined alcohol consumption at four quiz events in a private room of a pub. The impact of this intervention across a whole venue for a sustained period of time remains unknown.

The current study is designed to estimate the impact of two distinct interventions to modify the range of serving sizes for beer and wine respectively on alcohol sales across whole venues over a period of four weeks. In Intervention 1, licensed premises will add a two-thirds pint option to their existing range of serving sizes for beer and cider sold on tap. In Intervention 2, licensed premises will remove their largest serving of a glass of wine – either 250ml or 175ml. The primary outcomes assess the specific effects of each intervention on sales of beer and cider for Intervention 1 and sales of wine for Intervention 2. The effects of the interventions on sales of other alcoholic drinks will also be assessed.

A pilot study was conducted in four licensed premises (‘sites’) using an ABA reversal design conducted over three four-week periods. All sites completed baseline periods of “business as usual” (A) before and after the intervention period (B) in which they implemented Intervention 1. Two of these sites additionally implemented Intervention 2 at the same time. The pilot showed that it was feasible to implement the study procedures and collect the required data.

# Aim

The aim of this study is to estimate the impact of modifying the range of serving sizes for alcohol on alcohol sales.

# Methods

## Study design

The study has a reversal design with three consecutive four week periods as follows: ABA, where A represents the baseline periods during which standard serving sizes will be served and B represents the intervention periods in which the range of serving sizes will be modified.

Sites agreeing to implement both interventions will first complete intervention 1 followed by intervention 2 or vice versa.

## Interventions

### Intervention 1: Adding a two-thirds pint serving size

Sites will add a two-thirds pint serving size for all beers and ciders available on tap. The two-thirds pint will be offered in addition to the existing one pint and half-pint serving sizes, with proportionate pricing as far as is possible *i.e.* with a price which is linear-by-volume between the pint and half-pint sizes. Sites will choose two-thirds pint glasses to fit with their existing glassware, to be provided by the researchers. Bars will be allowed to keep the glassware after the study.

As part of the intervention, sites will adopt a range of strategies in keeping with their existing practices to draw customers’ attention to the new two-thirds pint option, including information on menus, signs behind the bar and prompting by bar staff when customers are ordering their drink. These practices will be described but not controlled.

### Intervention 2: Removing the largest serving size for glasses of wine

Sites will reduce their range of serving sizes for glasses of wine by removing the largest serving size for a glass of wine in their existing range. This will either be 250ml or 175ml, with 125ml sizes always available in keeping with current regulations for selling alcohol in licensed premises. Menus and signage will be updated to reflect this change.

Within the TIPPME intervention typology for changing environments to change behaviour (Hollands et al., 2017), the type of intervention proposed here is ‘size’, and is focused on the product itself *i.e.* alcohol (as opposed, for example, to aspects of the wider environment).

## Participants and recruitment

A minimum of 14 sites will be recruited to intervention 1. Sites will be a mixture of bars in university settings, non-student bars, restaurants and pubs. A minimum of five non-student bars will be recruited to intervention 2 due to greater variability observed at student bars during the pilot study.

Financial compensation for participating in this study is outlined below but subject to change if recruitment targets are not met, with the option of increasing the initial offer by 15% i.e. by £37.5 (plus 20% VAT) for Intervention 1 and £150 (plus 20% VAT) for Intervention 2. Sites will be reimbursed for the costs of any necessary changes to menus, signs or glassware.

Intervention 1:

ABA: £300 inclusive of VAT and glassware

Intervention 2:

ABA: £1200 inclusive of VAT

### Inclusion criteria:

- Sell beer (and/or cider) on tap in sizes larger than two-thirds of a pint (Intervention 1)
- Sell a minimum of 150 pints of beer and/or cider on average per week (Intervention 1)
- Willing to introduce a two-thirds pint serving size for all beer and cider available on tap at a proportionate price (Intervention 1)
- Sell wine by the glass in serving sizes greater than 125ml (*i.e.* 175ml or 250ml) (Intervention 2)
- Sell a minimum of 100 glasses of wine on average per week (Intervention 2)
- Willing to cap the serving size of a glass of wine (Intervention 2)
- Have an electronic point of sale (EPOS) till system to record daily sales of all drinks and their served sizes.
- Are primarily indoor, permanent establishments in a fixed location; i.e. not purposefully temporary or time-limited (e.g. pop-up), or mobile venues (e.g. vans)

### Sample size determination

#### Intervention 1

Simulations based on data from the pilot study of four sites – one restaurant and three student bars – suggested that 14 sites using an ABA design with each period lasting four weeks would provide 81% power to detect a predicted effect of 8.4% reduction in log beer volume. At least five of the bars recruited to this intervention will be non-student bars given the anticipated greater variability of data from student bars, as observed at two student bars in the pilot study. This will ensure a reasonable spread across different kinds of establishments.

#### Intervention 2

Simulations based on data from the pilot study of two sites – both student bars - using an ABA design with each period lasting four weeks suggested that 87 would need to be recruited to provide at least 80% power to detect a predicted effect of -226.8ml. It is not feasible to recruit this number of sites and this estimate is very uncertain. The current study is being conducted in a minimum of five bars which is achievable within available resources. At least five of the bars recruited to this intervention will be non-student bars to avoid the problem of widely variable data from the student bars found in the pilot study. Accordingly, although expecting only low power we consider this to be an opportunistic study providing preliminary evidence to inform future research, including more precise calculation of likely effect size and required sample size for future studies.

### Withdrawal of participants

Site leads will be informed that they are free to withdraw from the study at any time. In the event that a bar withdraws from the study during data collection, the investigator retains the ability to use all information received prior to withdrawal unless the site lead requests that the data are deleted.

# Measures

## Primary outcome

Intervention 1: Daily volume (in ml) of beer and cider purchased (including on tap, bottle or can), measured using electronic records of sales.

Intervention 2: Daily volume (in ml) of wine sold (including by the glass, bottle and carafe), measured using electronic records of sales.

## Secondary outcomes

1. Daily volume of beer and cider sold by serving size (pint, ½ pint, 2/3 pint, bottle sizes, can sizes)
2. Daily volume of wine purchased by serving size (125ml, 175ml, 250ml, carafe, 750ml bottle)
3. Daily volume of alcohol sold excluding beer and cider (Intervention 1) or wine (Intervention 2)
4. Daily revenue from food and alcoholic and non-alcoholic drinks
5. Daily number of transactions
6. Daily amount of beer and cider (Intervention 1) or wine (Intervention 2) sold in units of alcohol (in cases where it is practical to extract this information from electronic sales data).

## Additional measures

For sites implementing Intervention 2, the capacity of their usual wine glasses will be recorded.

# Procedure

Ethics approval has been obtained from University of Cambridge Psychology Research Ethics Committee (PRE.2019.035) and this protocol will be pre-registered on the Open Science Framework.

Sites will change their available serving sizes for beer and cider or wine two times over a period of 12 weeks.

Till systems will be updated as appropriate to reflect the new serving sizes.

Site leads will be contacted one day before each reversal to remind them of the required changes. Fidelity to the protocol for Intervention 1 will be checked either remotely or by site visits organised by the research team in the first days after each reversal. The method used for Intervention 1 will be determined by available resources. For Intervention 2, due to the difficulty inherit in remotely assessing fidelity to the protocol, these checks will all be conducted by site visits (See Appendix A for the fidelity check protocol).

Site leads and staff will be issued with a simple explanation to give to patrons who ask why serving sizes have changed: “We have been receiving requests for differently sized drinks so we are trying out some changes for a few weeks”.

Site leads will be invited to take part in a 30-minute end-of-study interview after the final baseline period to answer questions about their experience of taking part (Appendix B). This will take place by telephone or face-to-face.

Prior to publication, results will be shared with the site leads by telephone or face-to-face and they will be invited to comment.

# Data analysis

Two regression analyses will be used to predict the daily volume of beer and cider (Intervention 1) or wine (Intervention 2) sold during each four-week period of the study. Analyses will include dummy variables such as day of the week, overall daily sales at the site, and the maximum daily local temperature. Similar analyses will be used to investigate the secondary outcomes.

Dummy variables will be considered for use on occasions where there are unusual patterns of drinking caused, for example, by major sporting events or beer festivals.

Basic summary statistics will be presented individually and overall. An overall effect of each intervention will be estimated using regression, and a model simultaneously estimating all the individual site effects.

# Research governance

Research will adhere to the Wellcome Trust Policy on Good Research Practice and the UK Policy Framework for Health and Social Care Research. Researchers also follow the principles laid out in the UUK concordat to support research integrity.

## Ethical considerations and informed consent

Ethics approval has been obtained from the Cambridge Psychology Research Ethics Committee based at the University of Cambridge (PRE.2019.035). The investigator will provide the site leads with an information sheet explaining the nature, purpose and risks of the study. The site leads will be given sufficient time to read the information, consider any implications, and raise any questions with the investigators prior to making a decision to participate. Written consent will then be obtained. The site leads will be informed that they are free to withdraw from the study at any time.

## Sponsorship

The study will be sponsored by the University of Cambridge.

## Insurance

The study will be insured by the University of Cambridge. The University of Cambridge arranges insurance cover for legal liability to pay damages for injury to volunteers participating in the study which has been caused by the University or its employees. Adequate provision is made for insurance or indemnity to cover liabilities which may arise in relation to the design, management and conduct of the research project.

## Safety

The sites will follow their usual safety procedures. It is possible that the intervention increases alcohol sales amongst patrons, contrary to the study hypothesis. Local licensing regulations require the licensee to abide by the terms of the license, which includes not serving alcoholic beverages to a drunk person.

## Incident reporting

Incident reporting will follow the usual process for the site and University of Cambridge procedures. Incidents will be documented as soon as possible by the site manager using an incident report. The reports will be anonymised by unique study identifier and stored in a locked filing cabinet. Incidents will be followed up until resolved if possible. At the end of the study a safety report will be compiled and sent to the Principal Investigator listing all incidents. The Cambridge Psychology Research Ethics Committee will be notified of breaches as appropriate.

## Data management

All aspects of the General Data Protection Regulation, Data Protection Bill and the Freedom of Information Act 2000 will be adhered to. All personal data will be treated as confidential.

### Personally Identifiable Data (PID)

The University of Cambridge is the data controller for this study. Hard copy consent forms will be stored in a locked filing cabinet for one year after study completion, after which these documents will be destroyed. PID including the bar manager names and contact details will be stored on the Cambridge Secure Data Hosting Service (SDHS).

### Anonymous study data

Electronic data will be anonymised by a unique study identifier and the key located on the hard copy consent forms.

Anonymous sales data will be provided by the sites under a Data Transfer Agreement. Expectations for the Data Transfer Agreement are set out in the remainder of this section, although terms may vary as part of the negotiation process. A unique study identifier will be given to each site (with the key held on the hard copy consent form).

Anonymous study data will be stored on University of Cambridge network drives if electronic and/or in locked filing cabinets. Computer data files will be regularly backed up on a University of Cambridge network drive.

### Data sharing

Anonymous study data may be shared with collaborators for the purposes of analysis and results interpretation under appropriate collaboration agreements.

### Long term data archiving

At the end of the study, electronic study data (including finalised anonymous data sheet) will be transferred to a designated storage facility for long-term archiving. Hard copy data will be retained in locked storage facility. Study data will be kept for a minimum of 20 years.

Collaborators may retain anonymous study data in line with the relevant collaboration agreements.

### Open access

The data from this study will not be made available open access due to commercial sensitivity.

### Revoked data

If a site lead decides that they do not want their site’s data used after participation they can request that the data are withdrawn. They can request this up to one year after study completion.

### Quality control and quality assurance

The investigators will be responsible for data quality.

# Publication policy

The findings from this study will be published in at least one scientific journal and made available open access. They will also be presented at one or more scientific meetings.

# Study personnel

Eleni Mantzari

Senior Research Associate

Behaviour and Health Research Unit

University of Cambridge

Institute of Public Health

Cambridge CB2 0SR

Tel: +44 (0)1223 (7)61513

Email: [ikl22@medschl.cam.ac.uk](mailto:ikl22@medschl.cam.ac.uk)

Emily Pechey

Research Assistant

Behaviour and Health Research Unit

University of Cambridge

Institute of Public Health

Cambridge CB2 0SR

Tel: +44 (0)1223 (7)61513

Ilse Lee

Research Assistant

Behaviour and Health Research Unit

University of Cambridge

Institute of Public Health

Cambridge CB2 0SR

Tel: +44 (0)1223 (7)61513

Katie De-loyde

Research Associate (Statistician)

Tobacco and Alcohol Research Group

School of Psychological Science

University of Bristol

12a Priory Road

Bristol BS8 1TU

Tel: +44 (0) 117 92 88011

Email: [kd16662@bristol.ac.uk](mailto:kd16662@bristol.ac.uk)

Mark Pilling

Senior Research Associate in Statistics

University of Cambridge

Institute of Public Health

Cambridge CB2 0SR

Email: [mark.pilling@medschl.cam.ac.uk](mailto:mark.pilling@medschl.cam.ac.uk)

Gareth J. Hollands

Senior Research Associate

Behaviour and Health Research Unit

University of Cambridge

Institute of Public Health

Cambridge CB2 0SR

Email: [gjh44@medschl.cam.ac.uk](mailto:gjh44@medschl.cam.ac.uk)

Theresa M. Marteau

Professor, Director

Behaviour and Health Research Unit

University of Cambridge

Institute of Public Health

Cambridge CB2 0SR

Email: [tm388@medschl.cam.ac.uk](mailto:tm388@medschl.cam.ac.uk)

# Funding Source

Collaborative Award in Science from Wellcome Trust (Behaviour Change by Design: 206853/Z/17/Z) awarded to Theresa Marteau, Paul Fletcher, Gareth Hollands and Marcus Munafò. The funder will not be involved in the study design or data analysis.

# Conflicts of interest

There are no known conflicts of interest to declare.

# References

Burton, R., Henn, C., Lavoie, D., O’Connor, R., Perkins, C., Sweeney, K., … Sheron, N. (2017). A rapid evidence review of the effectiveness and cost-effectiveness of alcohol control policies: an English perspective. *The Lancet*, *389*(10078), 1558–1580. https://doi.org/10.1016/S0140-6736(16)32420-5

Codling, S., et al. (2020). "Impact of bottle size on in‐home consumption of wine: a randomized controlled cross‐over trial." Addiction (Abingdon, England) **115**(12): 2280.

Gakidou, E., Afshin, A., Abajobir, A. A., Abate, K. H., Abbafati, C., Abbas, K. M., … Murray, C. J. L. (2017). Global, regional, and national comparative risk assessment of 84 behavioural, environmental and occupational, and metabolic risks or clusters of risks, 1990-2016: A systematic analysis for the Global Burden of Disease Study 2016. *The Lancet*, *390*(10100), 1345–1422. https://doi.org/10.1016/S0140-6736(17)32366-8

Hollands, G.J., Shemilt, I., Marteau, T. M., Jebb, S. A., Kelly, M. P., Nakamura, R., … Ogilvie, D. (2013). Altering Micro-Environments to Change Population Health Behaviour: Toward an Evidence Base for Choice Architecture Internventions. *BMC Public Health*, *13*(1), 1218.

Hollands, Gareth J., Bignardi, G., Johnston, M., Kelly, M. P., Ogilvie, D., Petticrew, M., … Marteau, T. M. (2017). The TIPPME intervention typology for changing environments to change behaviour. *Nature Human Behaviour*, *1*(8). https://doi.org/10.1038/s41562-017-0140

Hollands, Gareth J., Shemilt, I., Marteau, T. M., Jebb, S. A., Lewis, H. B., Wei, Y., … Ogilvie, D. (2015). Portion, package or tableware size for changing selection and consumption of food, alcohol and tobacco. *Cochrane Database of Systematic Reviews*. https://doi.org/10.1002/14651858.CD011045.pub2

Kersbergen, I., Oldham, M., Jones, A., Field, M., Angus, C., & Robinson, E. (2018). Reducing the standard serving size of alcoholic beverages prompts reductions in alcohol consumption. *Addiction*. https://doi.org/10.1111/add.14228

Pilling, M., et al. (2020). "The effect of wine glass size on volume of wine sold: a mega‐analysis of studies in bars and restaurants." Addiction **115**(9): 1660-1667.

# Appendix A: Fidelity check protocol

Fidelity checks will take place at each site in the first days after each change is made according to the protocol (*i.e.* in the first days after a site has introduced a 2/3 pint (intervention 1) or removed the largest serving for a glass of wine (intervention 2) and in the first days after the 2/3 pint should be removed or the largest serving for a glass of wine reintroduced) to ensure the study protocol is being implemented correctly.

*Intervention 1*

Checks will be made either during a visit by a confederate or remotely, depending on available resources.

**During visits**, compliance with the intervention will be checked as described below.

*Introduction of 2/3pint:*

1. A confederate will order a 2/3 pint of any beer or cider. Compliance with the intervention would require bar staff to serve a 2/3 pint of the chosen beverage.
2. A confederate will observe the site for a period of 30 minutes. Compliance with the intervention would require that customers asking for a 2/3 pint are being served 2/3 pints.
3. The bar area will be checked to ensure there is signage informing of 2/3 pint options. Printed and/or electronic menus will also be checked to ensure 2/3 pint options are included
4. A confederate will order a half pint of any beer or cider. Compliance with the intervention would require bar staff to not mention the 2/3 pint option.

*Removal of 2/3 pint:*

1. A confederate will order a 2/3 pint of any beer or cider. Compliance with the intervention would require bar staff not to serve a 2/3 pint of the chosen beverage.
2. A confederate will observe the site for a period of 30 minutes. Compliance with the intervention would require that customers asking for a 2/3 pint are not being served 2/3 pints.
3. The bar area will be checked to ensure any signage informing of 2/3 pint options has been removed. Printed and/or electronic menus will also be checked to ensure 2/3 pint options are removed

Remote checks will be made using a video conferencing application. A meeting will be arranged with the bar manager/representative during which members of the research team will request verbal confirmation that the intervention has been implemented or removed and ask to see the changes made to the bar area to accommodate the introduction or removal of 2/3 pints (i.e. signage, menu changes, relevant glasses on display). Compliance with the intervention would require verbal and visual confirmation that all necessary changes have been made. The research team will then remotely observe the bar area for 15 minutes during service time, if feasible. Compliance with the intervention would require that customers asking for a 2/3 pint are being served 2/3 pints.

*Intervention 2*

Checks will be made during a visit by a confederate.

.

*Removal of the largest serving for a glass of wine:*

1. The confederate will order a large glass of wine. Once the glass of wine is served, the confederate will ask the staff member to verify the measure that is being served. Compliance with the intervention would require staff to serve only up to the maximum serving size for a glass of wine that can be offered during the intervention (to be agreed before the study starts with the study team).
2. The confederate will observe the site for a period of 30 minutes. Compliance with the intervention would require that customers ordering wine are not provided with a serving size greater than that which had been agreed before the study.
3. Printed and/or electronic menus will also be checked to ensure the largest serving size of wine has been removed

*Reintroduction of the largest serving for a glass of wine:*

1. The confederate will order a large glass of wine. Compliance with the intervention would require staff to serve the maximum serving size for a glass of wine that was available before the study (to be agreed before the study starts with the study team).
2. The confederate will observe the site for a period of 30 minutes. Compliance with the intervention would require that customers ordering large glasses of wine are being served the largest glasses that were available before the study.
3. Printed and/or electronic menus will also be checked to ensure the largest serving size of wine has been reintroduced

In all cases, a site will be considered to have failed the fidelity check if either of the criteria A or B are not satisfied. If criterion C is not satisfied, this will be recoded but will not result in a site failing the fidelity check.

If a site fails any of the checks they will be asked to rectify the observed protocol violation before an additional fidelity check takes place within 24 hours. A site might be asked to extend the study period to make up for days that have to be excluded from the analyses due to failed fidelity checks.

# Appendix B: End of study interview

This interview schedule is designed for the site leads who participated in ‘Modifying the range of serving sizes in bars and restaurants to reduce sales of alcohol: a field study’. The interview will be conducted over the phone or in person.

## The interview

*This conversation will last about 30 minutes. I will ask you some questions about your thoughts and experiences of taking part in the study. This interview will be tape recorded to ensure we can accurately capture your responses. Is this ok?*

1. Why did you decide to take part in the study?
2. What did you think of the changes you made to your serving sizes of beer and cider?
   1. Did you experience any practical problems with making the change? If so, what were they?
   2. How did staff deal with the change to your serving sizes?
   3. Did you notice any staff down-selling from pints to two thirds or up-selling from halves to two thirds?
   4. What was the reaction from customers to the two-thirds pints? Did you receive any complaints?
   5. Do you think the two-thirds pints changed how much beer and cider your customers ordered and drank?
   6. Do you have any plans to introduce two-third pints permanently?
3. What did you think of the changes you made to your serving sizes of wine?
4. Did you experience any practical problems with the change? If so, what were they?
5. How did staff deal with the change?
6. Did you notice any changes in the way staff dealt with customers ordering glasses of wine?
7. What was the reaction from customers to removing the largest serving for a glass of wine? Did you receive any complaints?
8. Do you think changing the serving sizes changed how much people ordered and consumed?
9. Do you have any plans to permanently remove your largest serving for a glass of wine?
10. How would you feel about the sales data you provided us being made available publically as ‘open data’? (This data would remain anonymous)
11. Finally, do you have any other comments about this study or the serving sizes of alcohol?
12. Would you consider taking part in a future study?

Many thanks.
