# Supplementary material for: Impact on wine sales of removing the largest serving size by the glass: An A-B-A reversal trial in 21 pubs, bars, and restaurants in England
Source: PLoS Med. 2024 Jan 18;21(1):e1004313. doi: 10.1371/journal.pmed.1004313 (PMC10796003; doi:10.1371/journal.pmed.1004313)
Supplement: S1 Statistical analysis plan — (DOCX) [file pmed.1004313.s003.docx]

# ANALYSIS PLAN for Intervention 2 (wine study)

The impact of altering serving sizes of wine on alcohol consumption: a field study

Eleni Mantzari, Emily Pechey, Ilse Lee, Katie De-loyde, Mark Pilling*,* Gareth J. Hollands, Theresa M. Marteau

# Study aim

The aim of this study is to estimate the impact of modifying the range of serving sizes for alcohol on alcohol sales.

# Hypotheses

Removing the largest available serving of wine by the glass (250ml or 175ml) reduces the volume of wine sold.

## Sample size

Simulations based on data from a pilot study of four sites – one restaurant and three student bars- using an ABA design with each period lasting four weeks suggested that 87 sites would need to be recruited to provide at least 80% power to detect a predicted effect of -226.8ml. It is not feasible to recruit this number of sites and this estimate is very uncertain. The current study was initially planned with a minimum of five bars, all of which would be non- student bars to avoid the problem of widely variable data from the student bars found in the pilot study. Based on available resources recruitment was increased to 21 sites. Although power to detect possible effects is still expected to be low, we consider this to be an opportunistic study providing preliminary evidence to inform future research.

# Study design

The study has a reversal design, with three consecutive four week periods as follows: ABA, where A represents the baseline periods during which standard serving sizes are served and B represents the intervention periods in which the range of serving sizes is modified.

Control period (A): Wine served in the site’s usual serving sizes for four weeks (i.e., usual practice).

Intervention period (B): Removing the largest serving size for glasses of wine. This was 250ml for 18 sites and 175ml for 3 sites.

## Primary outcome

Daily volume (in litres) of all wine purchased, regardless of serving size (including by the glass, bottle and carafe), measured using electronic records of sales.

## Secondary outcomes

Four secondary outcomes will be recorded from electronic records of sales:

- - Daily volume (in litres) of wine sold by each serving size, including but not limited to the following sizes:

- 125ml

- 175ml

- 250ml

- 500ml carafe

- 750ml bottle

- Daily volume (in litres) of beer and sider sold
  - Daily revenue from food, alcoholic and non-alcoholic drinks
  - Daily number of transactions, where available

## Additional measures

Maximum daily local temperature. Special events (e.g. Bank Holidays, other holidays, major sporting events, freshers’ week etc).

# Fidelity check

These were conducted twice at each site to establish whether the correct serving sizes were on offer at each site during the B period (intervention) and subsequent A period. Sites failing checks were informed, followed by a repeat fidelity check to establish whether the required change had occurred. Data from the weeks checks were failed were disregarded and periods were extended by the equivalent number of disregarded days, to ensure that each period always lasted four weeks.

# Data sharing

When data collection has ended, an Excel spreadsheet containing a clean data set will be sent directly to the study statistician completing the data analysis. A data dictionary will also be sent which includes all coding and ranges which exactly match the dataset.

# Recoding

Daily volume of wine sold in each serving size: This will be calculated by multiplying the number of items sold in each size by the volume of the serving size

Daily volume of all wine: This will be calculated my adding the total volume of items sold in each size.

Daily volume of beer and cider sold: This will be calculated by multiplying the number of items sold in each size by the volume of the serving size and adding all volumes together

For Sensitivity Analysis 4 the following will be calculated:

Mean daily sales for study site’s usual serving sizes (by adding the total volume together and dividing by the number of days the venue was open for in the A period).

Mean daily sales for study site’s when a new serving size is introduced (add the total volume together and dividing by the number of days the venue was open for in the B period).

# Outliers

Any outliers for the primary outcome will be identified using range checks, scatter plots and histograms on daily data. If any outliers are identified (defined with respect to the median absolute deviation), additional checks will be performed by the research team to ensure they are not the result of data entry errors.

Any events that may have taken place during the study period, which would impact alcoholic drinking levels (e.g., sporting events, freshers’ week, school holidays and bank holidays), will be noted by the research team to help understand possible outliers. These data will be presented in a table, split by site and intervention period, so that any differences in frequencies of these events can be assessed.

Any outliers (as defined above or caused by reported events that may have impacted drinking levels) will be included in the analysis but, if deemed necessary, the analysis will be run both with and without any true outliers to compare results.

# Missing data

Data that are missing due to an administrative or other error will be coded as -999. Any data that is deemed not applicable will be coded as -888.

# Missing data checks

If an excessive amount of missing data is identified for the primary outcome (>10%), the research team will be notified so that checks can be made.

Imputations will be made for small amounts of missing data. For example, if sites are unable to provide serving size information for their canned/bottles beers, this will be estimated based on the most common sizes available for online purchases.

# Violations of normality

It is expected that the primary and secondary outcomes of volume of sales and number of sales would follow a Normal distribution in the underlying population, and parametric analyses will be carried out accordingly. Nevertheless, whether the study data appears to follow a Normal distribution or not, the standard regression model diagnostics (such as normality plots of residuals) will be assessed, and as specified below alternative modelling used if appropriate.

If there is any indication of a strong departure from Normality for any of the outcomes, the p-value and 95% confidence interval (CI) will be calculated using the bootstrap method. If any outcome shows strong evidence of a positively skewed distribution, a transformation to another scale or will be considered.

# Descriptive statistics

All raw outcome data and additional data will be reported in tables with two columns comparing the mean in the A periods and the mean in the B periods (i.e., between the two serving size conditions). Percentages will be reported for categorical variables, and the mean and SD for continuous variables. This will be done individually for each site and combined across sites, for each intervention.

The mean difference (alongside the SD of the mean difference, and 95% confidence interval) between the A periods and the B periods, will also be reported for all outcomes, for each intervention.

Primary outcome data will be plotted for each intervention at each site, Wiine volume sold will be plotted against the study time periods, divided by site.

# Differences in A periods

Differences in the two A periods will be compared using a general linear model. Regardless of the results the statistical analysis plan will not change, but results may be interpreted differently if there are differences between the two A periods.

# Outcome analysis

All analyses will be conducted in either IBM SPSS v28 [3] or R v4[4]. Analyses will be coded in syntax and this will be added as Appendix A after the analyses are complete.

## Primary outcome analysis

### Primary analysis

For the primary analysis of the primary outcome, an analysis using general linear mixed model, will be used to predict daily volume of wine sales according to study Period (A vs B). Heteroscedasticity will be examined, to establish if both the mean and variance of volume of sales should be modelled. Site will be treated as a random factor. The Kenwood-Roger small sample adjustment to the degrees of freedom may be required.

An overall effect will be estimated from the appropriate general linear model. The mean difference and 95% CI for the mean difference and p-values will be presented. A Cohen’s d effect size and 95%CI will also be calculated.

Only sites that meet the following three conditions will be included in the primary analysis:

1. completed the study in full i.e. all 12 weeks
2. provided data for the full 12 weeks of the study
3. adhered to the protocol for intervention implementation i.e. their data did not suggest that the largest serving size of wine by the glass was waa sold during Period A.

**Sensitivity analyses**

To check the robustness of the primary conclusions from models when aggregating the three sets of four-week conditions, five sets of sensitivity analyses will be conducted:

1. Sensitivity analysis 1

Regression repeating the primary analysis while taking into account a number of covariates: *i)* the total number of special events in each period; ii) season at the start of the study (spring; summer; autumn; or winter); iii) maximum daily local temperature; iv) number of transactions. If numbers of transactions are not available for the majority of sites, total revenue will be used instead.

1. Sensitivity analysis 2

Regression using daily-level data from sites with incomplete data and whose data indicates a violation of protocol for intervention implementation i.e. sales of the largest serving size of wine by the glass during period B, to compare the mean daily volume of wine sales in the A period and the mean daily volume of sales in the B period.

1. Sensitivity analysis 3

A similar analysis to the primary analysis, but where we do not assume the first and second usual periods were the same (i.e. using A1, B & A2 levels).

1. Sensitivity analysis 4

Regression using period-level data to compare mean daily sales during Period A (aggregate value for 2 four-week A period) and mean daily sales during Period B (aggregate value for 1 four-week B period).

## Secondary outcome analysis

Two sets of secondary analyses will be conducted using similar analyses to above:

1. An analysis to predict daily volume of wine sales in each serving size (125ml, 175ml, 250m; 500ml carafe, 750ml bottle) according the study Period (A vs B).
2. An analysis to predict daily volume of beer and cider sold according the study Period (A vs B)
