# Supplementary material for: Impact on wine sales of removing the largest serving size by the glass: An A-B-A reversal trial in 21 pubs, bars, and restaurants in England
Source: PLoS Med. 2024 Jan 18;21(1):e1004313. doi: 10.1371/journal.pmed.1004313 (PMC10796003; doi:10.1371/journal.pmed.1004313)
Supplement: S1 Table — (DOCX) [file pmed.1004313.s004.docx]

**S4 Table:** Mixed effects GAM regression estimates (95% CI) for volume (ml) of wine sold per day (n=21) – intention to treat analysis

|  |  |  |  | **95% CI for estimate** | |
| --- | --- | --- | --- | --- | --- |
|  | **Estimate (SE)** | **t-value** | **P-value** | **Lower** | **Upper** |
| Intercept | 924.24 (203.60) | 4.54 | <0.001 | 525.2 | 1323.3 |
| Study period (ref: non-intervention) | -424.83 (130.12) | -3.26 | 0.001** | -679.9 | -169.8 |
| Day of the week_Tuesday (ref: Monday) | 543.49 (233.21) | 2.33 | 0.019* | 86.4 | 1000.5 |
| Day of the week_Wednesday (ref: Monday) | 835.98 (230.30) | 3.63 | <0.001** | 384.6 | 1287.4 |
| Day of the week_Thursday (ref: Monday) | 992.16 (233.38) | 4.25 | <0.001** | 534.7 | 1449.6 |
| Day of the week_Friday (ref: Monday) | 1173.81 (244.46) | 4.80 | <0.001** | 694.7 | 1652.9 |
| Day of the week_Saturday (ref: Monday) | 326.39 (241.02) | 1.35 | 0.176 | -145.9 | 798.8 |
| Day of the week_Sunday (ref: Monday) | 921.01 (234.43) | 3.92 | <0.001** | 461.5 | 1380.5 |
| Study Day | -2.69 (2.57) | -1.04 | 0.296 | -0.77 | 2.35 |
| Total revenue | 1.81 (0.046) | 38.33 | <0.001** | 1.72 | 1.90 |

*Significant at the p < 0.05 level; **significant at the p < 0.01 level. CI = confidence interval; SE = standard error.
