# Supplementary material for: Impact on wine sales of removing the largest serving size by the glass: An A-B-A reversal trial in 21 pubs, bars, and restaurants in England
Source: PLoS Med. 2024 Jan 18;21(1):e1004313. doi: 10.1371/journal.pmed.1004313 (PMC10796003; doi:10.1371/journal.pmed.1004313)
Supplement: S2 Table — (DOCX) [file pmed.1004313.s005.docx]

**S5 Table:** Mixed effects GAM regression estimates (95% CI) for volume (ml) of wine sold per day (n=20) – additional covariates

|  |  |  |  | **95% CI for estimate** | |
| --- | --- | --- | --- | --- | --- |
|  | **Estimate (SE)** | **t-value** | **P-value** | **Lower** | **Upper** |
| Intercept | 1874.1 (316.91) | 5.91 | <0.001 | 1252.9 | 2495.2 |
| Study period (ref: non-intervention) | -418.65 (131.15) | -3.19 | 0.001** | -675.7 | -161.6 |
| Day of the week_Tuesday (ref: Monday) | 531.31 (234.97) | 2.26 | 0.024* | 70.8 | 991.8 |
| Day of the week_Wednesday (ref: Monday) | 797.28 (232.19) | 3.43 | <0.001** | 324.2 | 1252.4 |
| Day of the week_Thursday (ref: Monday) | 895.46 (234.36) | 3.82 | <0.001** | 436.1 | 1354.8 |
| Day of the week_Friday (ref: Monday) | 1196.53 (247.73) | 4.83 | <0.001** | 710.9 | 1682.1 |
| Day of the week_Saturday (ref: Monday) | 346.01 (241.65) | 1.43 | 0.152 | -127.6 | 819.6 |
| Day of the week_Sunday (ref: Monday) | 906.82 (234.45) | 3.83 | <0.001** | 443.4 | 1370.2 |
| Study Day | -0.74 (2.64) | -0.28 | 0.778 | -0.59 | 4.43 |
| Total revenue | 1.84 (0.05) | 36.79 | <0.001** | 1.74 | 1.90 |
| Special events | -774.4 (260.57) | -2.97 | 0.003** | -1285.1 | -263.7 |
| Max daily temperature | -70.8 (15.7) | -4.50 | <0.001** | -101.6 | -39.9 |
| Study start seasons (ref: winter) | -6.13 (131.89) | -0.05 | 0.963 | -264.4 | 252.4 |

*Significant at the P < 0.05 level; **significant at the P < 0.01 level. CI = confidence interval; SE = standard error.
