# Supplementary material for: Impact on wine sales of removing the largest serving size by the glass: An A-B-A reversal trial in 21 pubs, bars, and restaurants in England
Source: PLoS Med. 2024 Jan 18;21(1):e1004313. doi: 10.1371/journal.pmed.1004313 (PMC10796003; doi:10.1371/journal.pmed.1004313)
Supplement: S3 Table — (DOCX) [file pmed.1004313.s006.docx]

**S6 Table:** Mixed effects GAM regression estimates (95% CI) for volume (ml) of wine sold per day (n=20) – separating non-intervention periods

|  |  |  |  | **95% CI for estimate** | |
| --- | --- | --- | --- | --- | --- |
|  | **Estimate (SE)** | **t-value** | **P-value** | **Lower** | **Upper** |
| Intercept | 843.8 (218.27) | 3.87 | <0.001 | 415.99 | 1271.60 |
| Intervention (ref: first non-intervention) | -463.19 (265.75) | -1.74 | 0.081 | -984.06 | 57.68 |
| Second non-intervention (ref: firs non-intervention) | -85.64 (464.69) | -0.18 | 0.854 | -996.41 | 825.13 |
| Day of the week_Tuesday (ref: Monday) | 540.14 (238.36) | 2.26 | 0.023* | 72.96 | 1007.32 |
| Day of the week_Wednesday (ref: Monday) | 831.19 (235.49) | 3.53 | <0.001** | 369.64 | 1292.7 |
| Day of the week_Thursday (ref: Monday) | 924.86 (239.09) | 3.87 | <0.001** | 456.41 | 1393.31 |
| Day of the week_Friday (ref: Monday) | 1157.41 (250.00) | 4.63 | <0.001** | 667.41 | 647.41 |
| N Day of the week_Saturday (ref: Monday) | 358.97 (246.10) | 1.45 | 0.145 | -123.39 | 841.32 |
| Day of the week_Sunday (ref: Monday) | 893.80 (242.38) | 3.68 | <0.001** | 418.75 | 1368.85 |
| Study Day | -0.33 (7.87) | -0.04 | 0.966 | -15.75 | 15.09 |
| Total revenue | 1.84 (0.04) | 38.79 | <0.001** | 1.75 | 1.93 |

*Significant at the p < 0.05 level; **significant at the p < 0.01 level. CI = confidence interval; SE = standard error.
